# Expression of Concern: Mechanism of Action of the Novel Nickel(II) Complex in Simultaneous Reactivation of the Apoptotic Signaling Networks Against Human Colon Cancer Cells

**DOI:** 10.3389/fphar.2016.00190

**Published:** 2016-06-15

**Authors:** Frontiers Editorial Office

**Affiliations:** FrontiersLausanne, Switzerland

With this notice, Frontiers states its awareness of serious allegations surrounding the article “Mechanism of Action of the Novel Nickel(II) Complex in Simultaneous Reactivation of the Apoptotic Signaling Networks Against Human Colon Cancer Cells” published on 28 January 2016. Our Chief Editors, Prof. Theophile Godfraind and Prof. Olivier Feron, will direct an investigation in full accordance with our procedures. The situation will be updated as soon as the investigation is complete.

